# An Infected, Noncoronary, Bare Metal Stent Presenting as a Right Groin Abscess

**DOI:** 10.1155/2023/6777086

**Published:** 2023-03-07

**Authors:** Madiha Khan, Youssef Mourad, Alan Zeitlin, Martine Alice Louis, Syed Ali Rizvi, Kelly L. Cervellione

**Affiliations:** ^1^New York Institute of Technology College of Osteopathic Medicine, 101 Northern Blvd., Glen Head, NY 11545, USA; ^2^Flushing Hospital Medical Center, 4500 Parsons Blvd., Flushing, NY 11355, USA

## Abstract

Bare metal stent infections complicating peripheral endovascular stenting are rare but can be associated with devastating morbidities. The current standard of care necessitates explantation and extra-anatomical bypass of the affected limb. We report the case of a patient presenting with a right groin abscess with draining sinuses secondary to an infected common femoral and right external iliac artery bare metal stent. In addition, a portion of the stent was explanted into the subcutaneous tissues instead of where it was placed intravascularly one year prior. The patient was not an ideal candidate for explantation and bypass due to significant medical comorbidities and underwent local debridement and long-term antibiotic management instead. His postoperative course was uncomplicated, and he had a successful outcome with management utilizing antibiotics and debridement. We aim to highlight the importance of recognizing bare metal stent infections along with their deceptive cutaneous manifestations in order to prevent the development of significant morbidity and mortality.

## 1. Introduction

Peripheral endovascular stenting and angioplasty (PTA) are used to treat peripheral vascular disease [1]. The more common complications related to PTA include hematomas, vessel thrombosis, distal embolization, arterial dissection, arterial rupture, stent displacement, neurological injury, and pseudoaneurysms. The incidence of vascular graft infections is relatively low, with estimates around 1.5% to 2% for most extracavitary grafts and up to 6% for grafts located in the groin [2]. Though rare, these infections can be associated with clinically significant morbidity or mortality especially because the infection may go unrecognized due to innocuous presentations such as an overlying skin abscess as in the case of our patient. The infection may not be apparent until it is more severe and extensive, potentially leading to septic shock. We report the case of a patient presenting with a right groin abscess with draining sinuses secondary to an infected and explanted common femoral (CFA) and right external iliac artery bare metal stent. This case illustrates the presenting features and management of this rare adverse event. Specifically, the innocuous presentation of an abscess or draining sinus in a patient with a history of vascular stents should be explored further to rule out stent complications. Early recognition and treatment can significantly improve a patient's clinical course and outcome.

## 2. Case Report

An 81-year-old male with a history of hypertension, chronic obstructive pulmonary disease, and atrial fibrillation status post right external iliac and common femoral artery stent placed in early 2021 presented in January 2022 with nontender erythema, edema of the right groin, and two sinuses with purulent drainage for four days. Of note, prior to stent placement, the patient also had a history of multiple admissions for septic shock in December 2020 and January 2021 secondary to urinary tract infection (UTI). For his current abscess, he was started on vancomycin at the nursing facility four days prior to presentation after an ultrasound of the area showed a well-circumscribed 2.8 × 1.7 × 0.8 cm nodule representing a reactive lymph node. Vital signs were all within the patient's baseline. Femoral, popliteal, and dorsalis pedis pulses on the left lower extremity were 2+. Sensory and motor function were intact. In the right lower extremity, there were absent femoral, popliteal, and pedal pulses. Both lower extremities were cutaneously viable without evidence of ulcerations or nonhealing wounds.

**Figure 1:**
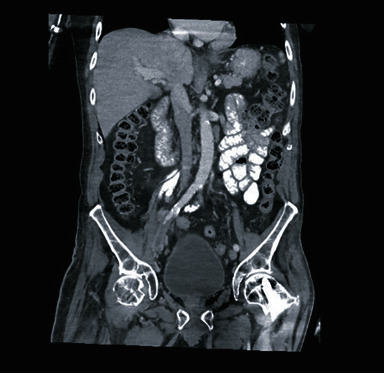
Computed tomography showing the vascular stent in the right external iliac artery and common femoral artery, surrounded by abnormal soft tissue density extending into the right inguinal subcutaneous fat and skin suspicious for infection.

**Figure 2:**
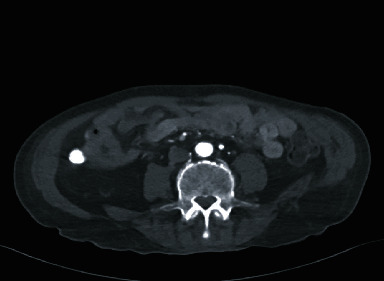
Computed tomography angiogram showing complete occlusion of the right external iliac artery with reconstitution of the common femoral artery at the groin; distal portion of the external iliac artery stent in subcutaneous tissue.

**Figure 3:**
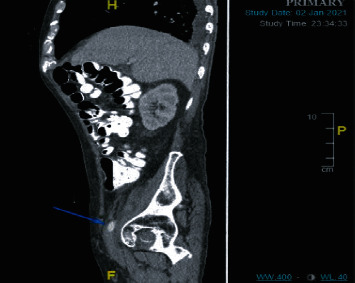
Computed tomography showing a small right common femoral artery pseudoaneurysm.

Upon workup, blood cultures demonstrated no growth. Computed tomography of the abdomen/pelvis (CTAP) with contrast during the current admission showed an abnormal soft tissue density surrounding the right external iliac artery. There was a vascular stent in the right common femoral artery, extending into the right inguinal subcutaneous tissues, with an intraluminal right external iliac artery thrombus proximal to the vascular graft (Figure 1). Computed tomography angiogram revealed that a distal portion of the external iliac artery stent was now located subcutaneously instead of where it was placed intravascularly one year prior in 2021 (Figure 2). Upon chart review, computed tomography scan from January 1, 2021, demonstrated a right common femoral artery pseudoaneurysm (Figure 3).

An incision and drainage of the right groin were performed in the operating room. The wound bed was copiously irrigated and packed with plain gauze. Explantation of the infected stent was held due to the significant comorbidities making the patient a poor surgical candidate. Wound cultures were positive for *Pseudomonas aeruginosa* and *Enterococcus faecalis*, and the patient was started on meropenem in addition to the continuation of vancomycin. He was monitored for two weeks after the incision and drainage and did well on the antibiotic regimen without complications. The patient was discharged back to the nursing facility with a midline for administration of meropenem until March 2022. Upon follow-up, our patient responded well to incision and drainage of the abscess, irrigation, and long-term antibiotics, without removal of the stent and revascularization. He continued to illustrate no signs of ischemia, ulceration, or nonhealing wounds and maintained intact sensory and motor function.

## 3. Discussion

Vascular graft infections are rare, occurring in 1.5% to 2% for most extracavitary grafts and less than 6% for grafts located in the groin [2]. Bare metal stents are used for the treatment of a plethora of diseases including coronary artery disease, renal artery stenosis, carotid artery stenosis, hemodialysis access sites, and lower extremity peripheral vascular disease [1]. As of 2022, fewer than 50 cases of bare metal stent infections have been reported in literature [3].

The common femoral artery access site, as in our patient, is the most frequently involved in the development of infection after percutaneous stenting [1]. Long-standing indwelling catheters or procedures involving repeated ipsilateral punctures, breach in sterility, insufficient skin preparation, prolonged time in surgery, puncture site hematoma, and multiple stents all increase the risks for graft infection [1]. Trauma to the endothelium of the artery during stent placement allows the stent to become a nidus for infection via exposed arterial media. Subsequent inflammation leads to necrosis and denudation of the arterial wall, the formation of a pseudoaneurysm leading to platelet adhesion and creation of a thrombus with septic emboli [1]. Corticosteroid use in patients can also contribute to this process [4]. Stent infection is confirmed by isolation of bacteria from either blood or perigraft drain fluid material [5].

The presenting symptoms of endovascular stent infection are nonspecific, but a draining sinus tract is highly suspicious for a graft infection although this visible cutaneous sign is rare as only two other cases exist in the literature regarding this finding [6, 7]. Although our patient had a draining sinus tract, the initial belief was that the skin lesion was due to an infected abscess as sinus tracts from infected stents are rare. Additionally, our patient was stable and afebrile, and cutaneous signs of limb ischemia were not present. The lack of substantial pulse in the right lower extremity was what prompted CT imaging, helping to finalize the diagnosis, luckily within a day of patient presentation. In our case, a pseudoaneurysm was noted on a prior CT which could have acted as the bed for platelet aggregation.

Prompt diagnosis of stent infections is necessary because 50% of patients with infected endovascular stents also have a pseudoaneurysm which can result in life-threatening bleeding or limb-threatening ischemia [2]. Importantly, endovascular stent infection is associated with a high rate of morbidity (17.2–75.3%) and mortality (32.5%), further justifying the necessity of swift identification [3].

Classification of graft infection is based on time of presentation of symptoms after stent placement [5]. Low-grade infections, as in our patient, have a late-onset time and can present with nonspecific symptoms such as weakness, weight loss, and feelings of malaise. High-grade infections have an early-onset time and can present with symptoms including but not limited to fever, nausea, and vomiting and, in the case of aortoenteric fistulae, abdominal pain, lumbar pain, and rectal blood loss [5]. Stent infections that are near skin level can present with reddened margins and purulent discharge [6]. In our patient, the cutaneous manifestations of the stent infection predominated without constitutional symptoms nor bleeding, characteristic of low-grade infection.

Imaging such as CT scans, angiography, ultrasonography, and tagged WBC scans can aid in diagnosing bare metal stent infections [1]. Computed tomography scan is gold standard in diagnosing stent infections. Ultrasound is less invasive but also less specific. MRI is comparable to CT scan in terms of reliability and is better at detecting smaller fluid collections [5]. [^18^F]Fluorodeoxyglucose-positron emission tomography (FDG-PET) scans can also detect inflammatory processes, however are only useful after a delayed time after operative placement due to the evolution of the healing process. Single-photon emission computerized tomography (SPECT) provides 3D imaging but has difficulty distinguishing physiologic from pathologic activity. Typically, a combination of CT scan and other modalities are used for a definitive diagnosis [5]. In our patient, an outpatient ultrasound prior to hospitalization showed a nonspecific inflammatory process. Computed tomography scan was diagnostic and revealed the exact location of the infectious process involving the CFA, the extent of infection, and the associated thrombosis.

Stent migration into the subcutaneous tissue can occur and proceed to external expulsion. The proposed mechanisms involve stent-graft thrombosis leading to infection or an immune reaction to the implant allowing development of the infection [5]. A similar case to ours has been previously reported in a 78-year-old male who suffered a stent-graft expulsion two years after the placement of a left superficial femoral artery stent. The patient exhibited purulent drainage from a cutaneous fistula and was managed with fistula drainage, systemic antibiotic therapy, and daily wound dressing with success at one-month follow-up [7]. Our patient had a similar presentation; however, his stent was located in the right external iliac and common femoral artery, which are deeper vessels than the superficial femoral artery described in the case above. Our case not only adds additional awareness regarding this rare presentation of stent expulsion but also demonstrates that stents located in deeper vessels can also exhibit this cutaneous presentation.

There is no consensus on specific treatment of infected bare metal stents [1]. Each case of an infected stent requires a unique surgical evaluation and approach to treatment. Indications for surgical intervention include mycotic aneurysm, recurrent septic emboli, persistent sepsis, or pseudoaneurysm formation. Good surgical candidates, for example, should undergo stent explantation with a bypass procedure. In many cases, the infected tissue must be removed, and the thrombosed vessel often requires revascularization [1]. The extraction of infected stents has been associated with a high degree of morbidity [1]. Autologous tissue is favored, but homografts as well as prosthetic material have been used [1]. Consensus is that extensive graft excision followed by reconstruction is the best method for treatment of infection whenever possible [5, 6].

However, these patients often present with multiple comorbidities and anatomic complexities that make removal difficult to justify as universal treatment, as was the case in our patient who had multiple comorbidities and was a poor surgical candidate for stent extraction. Additionally, poor results from radical excisions have been documented. Conservative treatment includes reestablishing vascularization using collateral blood vessels that are outside the field of infection (i.e., axillobifemoral bypass); however, this is not favored due to the fact that new surgical fields can become contaminated. Although higher mortality rates are reported with conservative treatment compared to graft excision and revascularization, risks versus benefit in every patient should be considered to determine the best course of action.

Additionally, microbes responsible for vascular graft infections have evolved with time. *Staphylococcus aureus* used to be the most common microorganism, but the expanded microbial spectrum now includes coagulase-negative staphylococci as well as *Pseudomonas aeruginosa* [2]. Antibiotic treatment, utilized along with debridement and wound care, was utilized in our patient and is another option for conservative treatment in patients who cannot undergo extensive vascular surgery such as ours; this approach can have successful outcomes as it did in our patient. Vancomycin and piperacillin-tazobactam are commonly prescribed along with rifampin to combat biofilm on a recently implanted stent [8].

Appropriateness of prophylactic antibiotics is debatable and should be considered on a case-by-case basis. It can be considered for diabetic, immunosuppressed, and cirrhotic patients [1]. Because of the history of COPD in our patient compromising his immune system, prophylactic antibiotics may or may not have been beneficial in preventing stent complications. Overall, however, our patient responded well to incision and drainage of the abscess, irrigation, and long-term antibiotics, specifically meropenem and vancomycin, without removal of the stent and revascularization. In future practice, it may be prudent to thoroughly consider prophylactic antibiotics in selecting patient populations such as the ones described above to decrease the significant morbidity and mortality associated with noncoronary bare metal stent infections. Furthermore, because of the possibility of these rare but significant complications, indications for placing stents should be as stringent as they are for bypasses.

## 4. Conclusion

Infections associated with endovascular stents are rare but carry the risk of high morbidity and mortality. Therefore, a high index of suspicion is needed. Patients should be educated on monitoring of the stent site to help identify infection early. Consideration of prophylactic antibiotics should be discussed on a case-by-case basis. Urgent surgical evaluation should be conducted when signs of infection such as wounds, draining sinuses, or fever are present. This case highlights the rare presentation of a draining fistula and abscess associated with an infection and expulsion of a bare metal stent. It additionally demonstrates the importance of a swift diagnosis to prevent morbidity and mortality as early intervention proved successful for our patient.
